# Extracellular acidosis enhances Zika virus infection both in human cells and ex-vivo tissue cultures from female reproductive tract

**DOI:** 10.1080/22221751.2021.1932606

**Published:** 2021-06-13

**Authors:** A. Varese, E. Dantas, A. Paletta, W. Fitzgerald, F. Di Diego García, G. Cabrerizo, F. Erra Diaz, L. A. Defelipe, H. Pallares, M. Dodes Traian, A. Gamarnik, J. Geffner, F. Remes Lenicov, L. Margolis, A. Ceballos

**Affiliations:** a Instituto de Investigaciones Biomédicas en Retrovirus y SIDA (INBIRS), Universidad de Buenos Aires (UBA) and Consejo Nacional de Investigaciones Científicas y Técnicas (CONICET), Ciudad de Buenos Aires, Argentina; b Section on Intercellular Interaction, Eunice Kennedy Shriver National Institute of Child Health and Human Development, National Institutes of Health, Bethesda, MD, USA; c Departamento de Química Biológica, Facultad de Ciencias Exactas y Naturales, Universidad de Buenos Aires, IQUIBICEN-CONICET, Ciudad de Buenos Aires, Argentina; d Fundación Instituto Leloir-CONICET, Buenos Aires, Argentina

**Keywords:** ZIKV, extracellular acidosis, sexual transmission, heparan sulphate, ex-vivo tissues

## Abstract

Zika virus (ZIKV) is a flavivirus transmitted by mosquitoes of the genus *Aedes*, but unlike other flaviviruses, ZIKV can be sexually transmitted by vaginal intercourse. The healthy vaginal pH ranges from 4.0 to 6.0, reaching values of 6.0–7.0 after semen deposition. Here, we report that low extracellular pH values (range 6.2–6.6) dramatically increase ZIKV infection on cell lines of different origin including some derived from the female genital tract and monocyte-derived macrophages. Furthermore, low pH significantly increased ZIKV infection of human ectocervix and endocervix cultured ex-vivo. Enhancement of infection by low pH was also observed using different ZIKV strains and distinct methods to evaluate viral infection, i.e. plaque assays, RT–PCR, flow cytometry, and fluorescence microscopy. Analysis of the mechanisms involved revealed that the enhancement of ZIKV infection induced by low pH was associated with increased binding of the viral particles to the heparan sulphate expressed on the target cell surface. Acidosis represents a critical but generally overlooked feature of the female genital tract, with major implications for sexual transmission diseases. Our results suggest that low vaginal pH might promote male-to-female transmission of ZIKV infection.

## Introduction

Zika virus (ZIKV) is an emerging pathogen associated with neurodevelopmental defects in foetuses, and a neurological syndrome in adults [1–4]. ZIKV is primarily transmitted by *Aedes aegypti* and *Aedes albopictus* mosquitoes, but sexual transmission has also been documented [5–10]. This has challenged the paradigm of arbovirus–host interactions opening new questions regarding the transmission of ZIKV infection.

Symptomatic ZIKV infection usually results in a mild and self-limiting febrile disease, although different reports have suggested a possible association with more serious conditions such as Guillain–Barré syndrome, and microcephaly in new-born infants from mothers infected with ZIKV during pregnancy [1–4]

Male-to-female, male-to-male, and female-to-male ZIKV sexual transmission have been reported [5–10]. Viral load in semen reaches titters up to 100,000 times higher than in blood and persists for up to six months after infection [11,12]. In addition, ZIKV in semen samples was shown to be able to productively infect Vero cells [13]. Moreover, a close association of ZIKV with the spermatozoa head has been reported [6].

Male-to-female transmission requires that ZIKV successfully resist vaginal acidosis [13,14]. Large areas of the mucosal surfaces of the genital tract are exposed to low pH values. The healthy vaginal environment is characterized by acidic values of pH between 4.0 and 6.0 [13,14]. In contrast, the pH of normal semen is slightly alkaline ranging from 7.2 to 7.8 [15]. Upon semen deposition, the vaginal environment can reach pH values ranging from approximately pH 6.0 to 7.0 for several hours after sexual intercourse [16,17], thus exposing ZIKV to a broad range of pH values.

Heparan sulphate (HS), a linear polysaccharide found in almost all tissues, has been identified as an important attachment factor for several viruses [18–28]. Previous studies have shown that ZIKV interacts with cell surface HS [29–32]. The interactions between some flavivirus, for example, dengue and yellow fever, and cell surface HS are required to concentrate virus particles at the cell surface and to facilitate binding to a second receptor specifically recognized by the viral envelope protein. Therefore, viral production was reduced when HS was desulfated or enzymatically removed from the cell surface [28].

ZIKV is the only flavivirus capable of being sexually transmitted, therefore, we hypothesized that ZIKV would be able to preserve infectivity under low values of extracellular pHs, such as those found in the female genital tract. Surprisingly, we found that acidosis not only preserves, but dramatically increases ZIKV’s ability to infect cell lines and ex-vivo tissue cultures derived from the female genital tract, as well as monocyte-derived macrophages (MDM) and primary astrocytes (NHA). Furthermore, we found that this enhancing effect is mediated by increased binding of viral particles to heparan sulphate (HS) expressed on the target cell surface.

## Materials and methods

### Viruses

ZIKV Senegal 1985 isolate (DAK-AR-41524_A1C1-V2, GeneBank accession no KX198134) was gifted by Dr A. Gamarnik. ZIKV Venezuela was isolated from a patient who travelled from Venezuela to Argentina in 2015 (strain ARCB116141, GeneBank accession no. MK637519) and gifted by Instituto Nacional de Enfermedades Virales Humanas “Dr Julio I. Maiztegui” (INEVH). ZIKV Puerto Rico 2015 Isolate (PRVABC59, GeneBank accession no KU501215) was gifted by Dr Viviana Castilla and the University of Buenos Aires, School of Sciences. ZIKV VR-1844 strain was acquired from ATCC. DENV-2 was a gift from Dr Andrea Gamarnik and was obtained by transfection of an *in vitro* transcribed RNA from the infectious DENV-2 cDNA clone pD2-IC 30-PA [33], originally isolated from a patient with dengue haemorrhagic fever (*Asian* genotype). All the viruses were propagated and titrated in Vero cells, and the infectious titer was expressed as pfu per ml. All infections were carried out using Senegal strain except where indicated.

### Cell lines and primary culture

VERO C1008 (clone E6, ATCC® CRL-1586™), HESC-T (ATCC® CRL-4003™) cells, and Normal Human Astrocytes (NHA, LONZA) were cultured in DMEM (Thermo Fisher) supplemented with 10% heat-inactivated foetal bovine serum (FBS, Thermo Fisher), penicillin (100 U/ml), and streptomycin (100 µg/ml). VK-2/E6E7 (ATCC® CRL-2616™), ECT-1/E6E7 (ATCC® CRL-2614™), and END-1/E6E7 (ATCC® CRL-2615™) cell lines were purchased from the American Type Culture Collection and cultured in Keratinocyte-Serum Free medium (GIBCO-BRL 17005-042) with 0.1 ng/ml human recombinant EGF, 0.05 mg/ml bovine pituitary extract, and additional calcium chloride 44.1 mg/l. Human monocyte was obtained from healthy donors by Percoll-Hypaque gradient and differentiated to monocyte-derived macrophages (MDM) with M-CSF for 5 days, or to monocyte-derived dendritic cells (DCs) with 10 ng/ml GM-CSF and 10 ng/ml of IL-4 for 5 days. Purity and differentiation were monitored by flow cytometry analysis prior to each experiment.

### Ex-vivo tissue culture of ectocervix and endocervix

Cervico-vaginal surgical pieces were obtained through the National Disease Research Interchange (Philadelphia, PA) according to IRB-approved protocols. Ecto- and endocervical tissues were dissected into 2-mm [3] blocks and incubated in 2 ml Eppendorf tubes (cat no. EP022363344) with ZIKV (VR-1844; moi 0.1) in DMEM adjusted to pH 7.3 or 6.2 for 1 h at 37°C. After two washes with PBS the tissue pieces were placed on top of Gelfoam pads sponges in DMEM with 10% FBS and incubated at 37°C as previously described [34].

### Cell viability assays

Apoptosis and necrosis were evaluated by staining with annexin-FITC/propidium iodide (BD Biosciences) by flow cytometry. Cells were labelled with annexin-V FITC and propidium iodide for 15 min at room temperature and analysed by flow cytometry using a BD FACSCanto cytometer and BD FACSDiva software (BD Biosciences). MTT reduction assay was performed on Vero cells following the manufacturer’s instructions (Merck).

### Viral infections and binding

Infections (moi = 0.1) were performed in serum-free RPMI (Thermo Fisher) supplemented with HEPES (10 mM) (Thermo Fisher) for 1 h at 37°C, 5% CO_2_, and pH values were adjusted using isotonic hydrochloric acid. After the infection, the supernatant was retrieved, pH values were reassessed, and cells were washed and placed in a culture medium. For binding assays, cells were incubated with the viral inoculum at different pH values at 4°C for 1 h and immediately after cells were washed four times with PBS, and RNA was extracted. NH_4_Cl treatment on Vero cells consisted of a pre-incubation of 1.5 h with 2, 20 and 100 mM of NH_4_Cl in RPMI 2% FBS. During the infection, pH was adjusted in RPMI with the corresponding concentration of NH_4_Cl. After 1 h cells were washed and infection was assessed after 48 h.

To abrogate HS synthesis, Vero cells were cultured for 72 h with 25 mM sodium chlorate in a custom-made Dulbecco modified Eagle medium lacking sulphate supplemented with 10% foetal calf serum, as described previously [35]. For enzymatic removal of HS, Vero cells were treated with 5 U/ml of a heparinase I and III blends (Sigma) for 2 h at 37°C.

### Intracellular pH measurement

Intracellular pH measurement was performed using BCECF-AM as previously described [36]. Briefly, Vero (5 × 10^6^ /ml) were loaded in PBS with 1 μg/ml BCECF-AM for 15 min at 37°C, washed in PBS, and resuspended in culture medium, adjusted to different pH values, in the absence or presence of NaHCO_3_. Immediately after, the values of intracellular pH for each condition were determined. The analysis was performed by flow cytometry, with excitation at 488 nm and emission analysis at FL1 and FL3. The intracellular pH was calculated from the ratio of emission intensities at the two wavelengths, standardizing by comparison with the fluorescence intensity ratios of cells whose intracellular pH values were fixed by incubation with nigericin (10 μM) in a high-potassium buffer.

### Viral detection by qPCR

Total RNA was obtained from 3 × 10^6^ previously washed cells lysed using a Pure-link Viral RNA DNA extraction kit following the manufacturer’s instructions (Thermo Scientific). Reverse transcription was carried out using Moloney murine leukaemia virus reverse transcriptase (Invitrogen) according to the manufacturer’s instructions. In brief, 5 µg of RNA were incubated for 50 min at 37°C in the presence of 200 ng random decamer primers (Invitrogen) and 10 mM dNTP mix. Quantitative real-time PCR was performed using SYBR Green PCR Master Mix (Invitrogen) in a 20-µl reaction. Both ZIKV and DENV genomes were detected using 200 nM of primers 5- GCCGCCACCAAGATGAACTGATTG -3 (forward) and 5- GCAGTCTCCCGGATGCTCCATC -3 (reverse). Reactions were carried out in StepONE Plus cycler (Thermo Scientific). The cycling programme used was 95°C for 10 min followed by 40 cycles of 95°C for 15 s and 60°C for 60 s. Data were analysed by the ΔΔCt method using GADPH as the reference gene.

### Luminex assay

When indicated, ZIKV infection was assessed measuring NS1 production by Luminex. Briefly, magnetic carboxylated microspheres (Luminex) were coated by two-step carbodiimide coupling with an antibody against NS1 (Fitzgerald, cat no. 10-2707). The standard curve was obtained by serial dilution of recombinant NS1 protein (Fitzgerald, cat no. 30-1931). For detection, an anti-NS1 antibody (Abcam cat no. 218546) was biotinylated using the EZ-Link Sulfo-NHS-Biotin (Thermo Scientific cat no. 21217). Streptavidin conjugated with R-Phycoerythrin (Thermo Scientific cat no. S866) was used to detect the biotinylated antibody. All experiments were acquired using a Bio-Rad Bio-Plex 200 System and analysed with the Bio-Plex Manager software.

### Flow cytometry and microscopy

For ZIKV detection rabbit pan-flavivirus anti-E (clone 4G2, Absolute Antibody) was used as a primary antibody and FITC conjugated anti-rabbit secondary was used for detection. Heparan sulphate (HS) expression was determined using the clone F58-10E4 (Amsbio) as a primary antibody and an anti-mouse IgM as a secondary antibody. Isotype-matched control antibodies were used, and a gate (R1) was defined in the analysis to exclude non-viable cells and debris, based on size and propidium iodine staining. Analyses were performed using a FACS flow cytometer (FACS CANTO I, Becton Dickinson) and the data were analysed using FlowJo software (Tree Star, Inc.).

For microscopy, Vero cells were fixed and permeabilized with methanol 20 min at 4°C, then stained with rabbit pan-flavivirus anti-E (clone 4G2, Absolute Antibody) as a primary antibody, and FITC conjugated anti-rabbit secondary was used for detection. Cells were mounted with Fluoroshield (Sigma Aldrich) containing DAPI and images were acquired using a confocal microscope (Zeiss Ism 880).

For Ki67 immunofluorescence (IF), Vero cells were fixated with 4% PFA and permeabilized with 0.3% Triton X-100. After blocking with 5% donkey serum (Sigma, cat.no. D9663-10ML) cells were stained with an anti- Ki67 (NB600-1252) as primary antibody and an anti-rabbit conjugated to Alexa-488 (Jackson ImmunoResearch) as a secondary antibody. The slides were mounted using a mounting medium with DAPI (Vector laboratories, cat no. H-1200-10). Images were acquired with a Nikon TI Eclipse microscope and quantified using QuPath 0.2.3.

### Statistics

Student paired *t*-test, One or two-way ANOVA were used to determine the significance of differences between groups. Multiple analyses were followed by Bonferroni’s multiple-comparison post-test. *P-*values < 0.05 were considered statistically significant.

## Results

### Low extracellular pH strongly enhances ZIKV infection.

**Figure 1:**
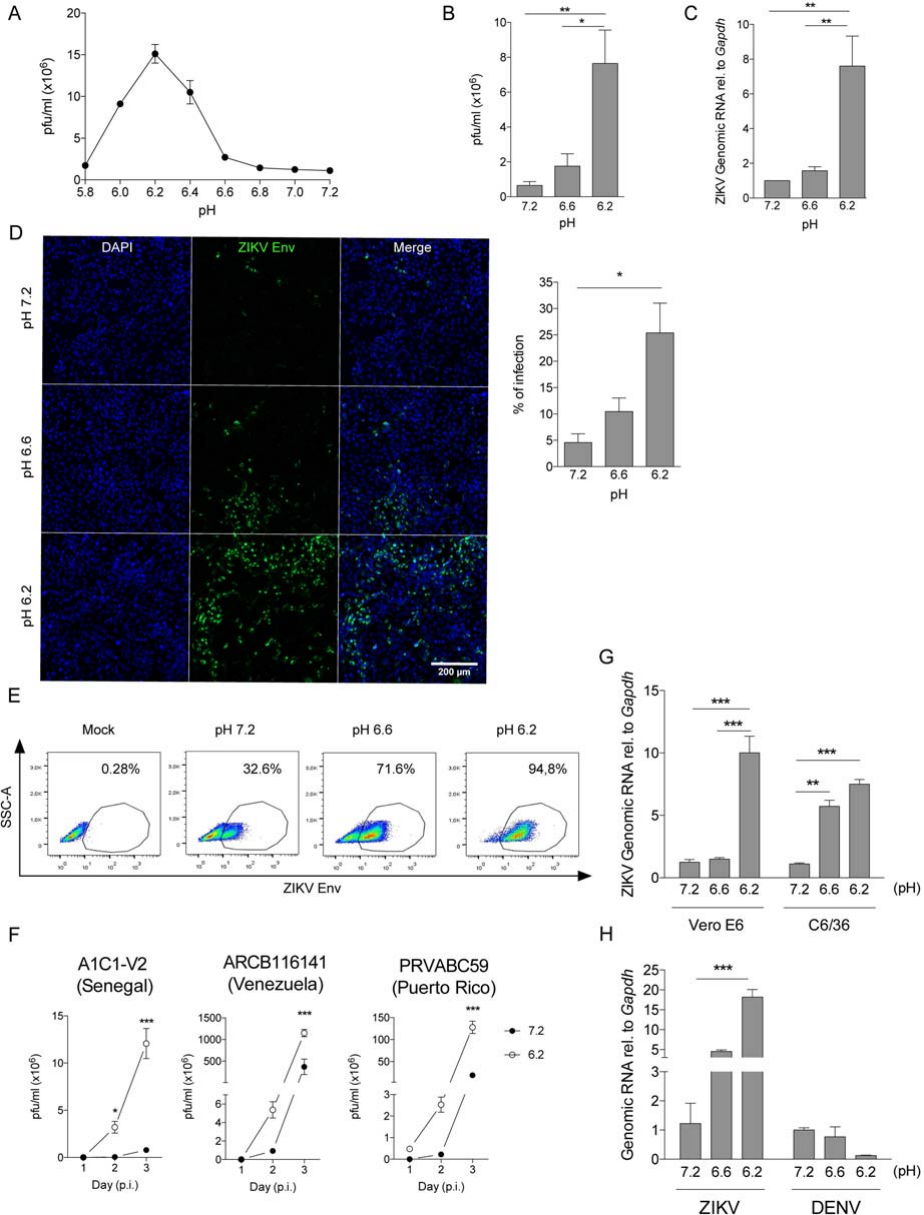
. Low extracellular pH enhances ZIKV infection of Vero cells. (A–E) Vero cells were incubated with ZIKV (A1C1-V2) for 1 h at different values of pH (moi 0.01). Then, cells were washed twice and ZIKV infection was analysed 48 h later in cell supernatants by pfu assays (A, B), qPCR (C), immunofluorescence (D), and flow cytometry using an anti-Env mAb (E). (F) Vero cells were incubated at pH 7.3 or 6.2 for 1 h at 37°C (moi 0.01) with different strains of ZIKV (Senegal, Venezuela or Puerto Rico isolates). Viral production was quantified in cell supernatants at days 1, 2 and 3 post-infection (p.i.). (G) Vero cells were incubated as described above with ZIKV (SEN) produced either in Vero cells or C6/36 cells. After 48 h, viral load was measured by qPCR. (H) Vero cells were incubated with ZIKV (moi 0.01) or with DENV-2 (moi 0.1), as described above. After 48 h, viral load was measured by qPCR. Data in A, D, E, F, G, and H are representative of three different experiments. Results in B and C were obtained in five independent experiments. Results are presented as mean ± SEM (B, C, D, G, H) and mean ± SD (A, F).

In the first set of experiments, we evaluated the impact of extracellular pH on ZIKV infection. Vero cells were incubated with ZIKV for 60 min in media adjusted to pH values of 7.2 (control), 7.0, 6.8, 6.6, 6.4, 6.2, 6.0, and 5.8; then cells were washed to remove the viral inoculum and a fresh medium was added (pH 7.3). Forty-eight hours later, the titters of infectious ZIKV were evaluated by plaque assays and expressed as plaque-forming units per millilitre (pfu/ml) (Figure 1(A)). Our results showed that pH values ranging between 5.8 and 6.6 markedly enhanced ZIKV infection, reaching a maximum effect at pH 6.2 (Figure 1(A,B). Similar results were observed when ZIKV infection was evaluated by NS5 ZIKV RNA expression (Figure 1(C)), fluorescence microscopy (Figure 1(D)), and analysis of intracellular ZIKV E protein expression by flow cytometry (Figure 1(E)). No changes in cell viability, metabolic activity (i.e. MTT reduction), and cell proliferation were observed in the range of pH tested (Supplementary Figure 1(A–C)).

We also analysed whether the higher infectivity observed at low pH was reproduced using different ZIKV strains: African isolate (Senegal, 1985) and American isolates (Venezuela and Puerto Rico, 2015). Productive viral infection was assessed by quantifying infectious virus in supernatants collected from day 1 to day 3 post-infection. All ZIKV strains analysed showed increased productive infection at pH 6.2 compared to pH 7.3 (Figure 1(F)). Similar results were also observed using viral stocks produced either in mosquito- or primate-derived cell lines (C6/36 and Vero respectively) (Figure 1(G)).

Next, we tested whether low pH might also increase the infection of the Dengue virus (DENV-2), which is closely related to ZIKV. Vero cells were inoculated in parallel experiments with ZIKV or DENV-2 at different values of pH for 60 min, washed, and cultured at pH 7.3 for 48 h. Then, viral RNA expression was measured by qPCR. We observed that low pH did not increase DENV infection (Figure 1(H)).

**Figure 2.**
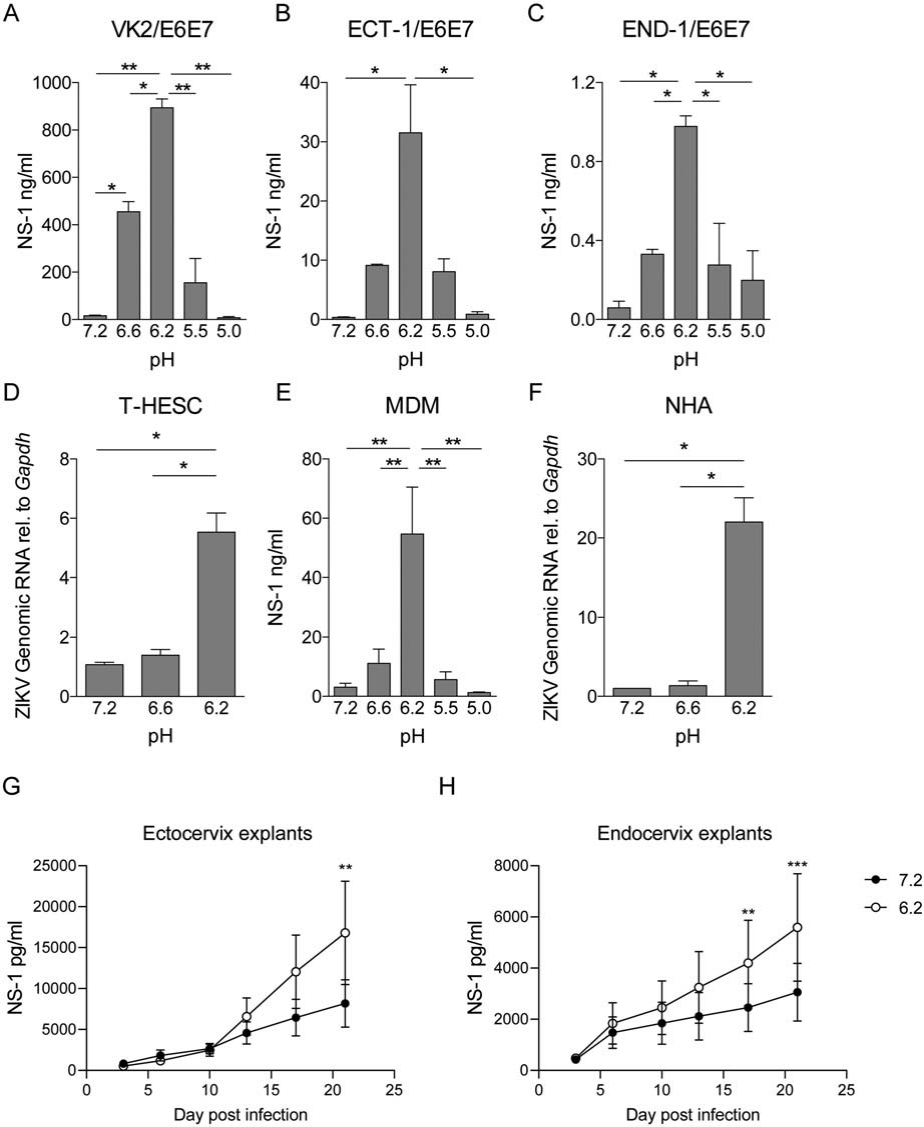
Acidosis enhances ZIKV infection in cells derived from the female reproductive tract and other target cells. Cells were incubated with ZIKV (A1C1-V2) at different pH values for 1 h at 37°C. Then, cells were washed and infection was assessed 72 h later. Infection of genital tract-derived cells VK2/E6E7 (A), Ect1/E6E7 (B), and End1/E6E7 (C) was quantified by measuring the extracellular concentration of NS-1 by Luminex. Infection of human endometrial fibroblasts (T-HESC) was quantified by qPCR (D). Infection of monocyte-derived macrophages (MDM) was quantified by measuring the extracellular concentration of NS-1 by Luminex (E). Primary culture-derived human astrocytes (NHA) infection was measured by qPCR (F). Human ectocervical (G) and endocervical (H) tissue cultures were dissected into 2 mm [3] pieces and infected with ZIKV (VR-1844 moi 0.1) either at pH 7.3 or 6.2 for 1 h at 37°C. The viral production was evaluated at days 3, 6, 10, 13, 17, and 21 by measuring ZIKV NS1 in the culture supernatant. Results in A, B, C, D, and F represent data of two independent experiments. Data in E comprises the results of four different donors. Panels H and G were constructed with data of three independent experiments per explant type, and data were analysed using paired two-way ANOVA. Results are presented as mean ± SEM.

To evaluate whether acidosis also increased ZIKV infection of cells derived from the female reproductive tract, vaginal (VK2/E6E7) ectocervical (Ect1/E6E7), and endocervical (End1/E6E7) cell lines and human-derived endometrial fibroblasts (T-HESC) were inoculated with ZIKV at pH values of 7.3, 6.6, and 6.2 for 1 h at 37°C. Cells were then washed, cultured for 48 h in fresh media (pH: 7.3), and evaluated for ZIKV infection. Figure 2(A–D) shows that infection was markedly increased at pH 6.2 compared to neutral pH. No changes in cell viability were observed in the range of pH tested (data not shown). Moreover, the enhancing effect of low pH on ZIKV infection was also observed using human monocyte-derived macrophages (MDM) and primary astrocytes (NHA) (Figure 2(E,F)). Of note, ZIKV infection enhancement by low pH is not reproduced in monocyte-derived dendritic cells (Supplementary Figure 2(A–C))

To demonstrate our hypothesis in a more physiologically relevant scenario, we performed experiments using a well-established technique for culturing and infecting ectocervical and endocervical tissues ex-vivo [34]. Human cervical tissues were dissected into 2 mm [3] pieces and infected with ZIKV either at pH 7.3 or 6.2 for 1 h at 37°C. The viral production was evaluated at days 3, 6, 10, 13, 17, and 21 by measuring ZIKV NS1 in the culture supernatant. Consistently, both endocervix and ectocervix explants produce a significant increase in viral titters when infected at pH 6.2, showing that the extracellular acidosis enhances ZIKV infection in tissues from the human female genital tract ex-vivo.

### Low extracellular pH increases ZIKV binding to target cells.

**Figure 3.**
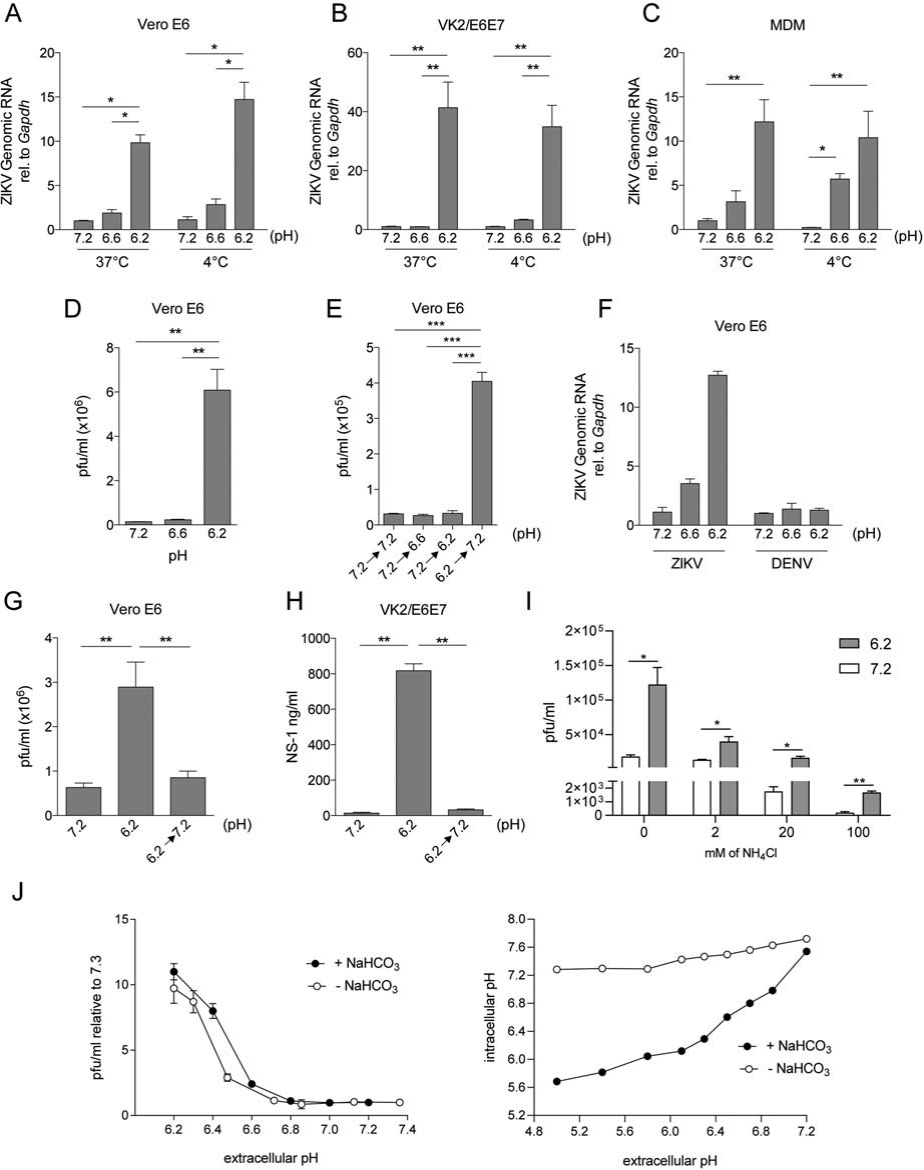
Low extracellular pH increases ZIKV binding to target cells. Vero cells (A), VK2/E6E7 cells (B), or MDM (C) were incubated with ZIKV (A1C1-V2; moi 0.01) for 1 h at 37°C or 4°C, at different pH values. Then, cells were washed four times and total RNA was extracted to determine ZIKV binding by qPCR. Vero cells were incubated at different pH values with ZIKV (moi 0.01) for 1 h at 4°C, at different pH values. Then, cells were washed and cultured at neutral pH for 48 h. Viral production was quantified by pfu (D). Vero cells were cultured with ZIKV (moi 0.01) at pH 7.3 for 1 h at 37°C, washed, and incubated for 1 h at different pH values. Then, cells were washed again and cultured for 48 h at pH 7.3. Viral production was determined by pfu (E). DENV-2 and ZIKV binding to Vero cells were assessed in parallel as described above (F). ZIKV suspensions were pre-incubated at pH 7.3 or 6.2 for 1 h at 37°C. Then, the viral suspensions at pH 6.2 were neutralized back to pH 7.3 (6.2→7.3) or not, and Vero (G) and VK2 cells (H) were infected with these viral suspensions at pH 7.3 or 6.2. (I) Vero cells were pre-incubated for 1.5 h with 2, 20 and 100 mM of NH_4_Cl. Then ZIKV infections were performed in pH adjusted media with 2, 20, or 100 mM of NH_4_Cl respectively. Viral production was evaluated 48 h post-infection by pfu assay. (J) Vero cells were infected for 1 h in pH adjusted NaHCO_3_-free or NaHCO_3_ rich DMEM. ZIKV infection was quantified by pfu 48 h after infection. In parallel, BCECF-AM pre-loaded vero cells were treated with pH adjusted bicarbonate-free DMEM and intracellular pH was determined by flow cytometry. Results are expressed as the mean ± SEM of three independent experiments.

We then evaluated whether the promotion of ZIKV infection induced by low pH was related to increased binding of viral particles to the target cell surface. In these experiments, Vero cells were incubated with ZIKV at pH values of 7.3, 6.6, and 6.2 for 1 h at 4°C or 37°C respectively. Then, cells were washed and cell-associated viruses were quantified by qPCR. We found that low pH markedly promoted the association of viral particles with Vero cells in assays performed at either 4°C or 37°C (Figure 3(A)). Similar results were observed in assays carried out with MDM or VK2/E6E7 cells (Figure 3(B,C)). These findings suggest that the enhanced binding of viral particles to the target cell surface, and no other early events that might occur at 37°C such as promotion of virus fusion, might be responsible for the enhanced effect induced by low pH on ZIKV infectivity. Supporting this, a marked increase in viral production was observed when Vero cells were incubated with ZIKV at pH 6.2 for 1 h at 4°C and after thoroughly washing they were cultured at neutral pH for 48 h (Figure 3(D)). By contrast, when Vero cells were incubated with ZIKV at pH 7.3 for 1 h at 4°C and after washing, cells were cultured for an additional hour at pH 6.2, washed again and cultured at neutral pH for 48 h, no promotion of infection was observed (Figure 3(E)). Interestingly, and in contrast to ZIKV, low pH did not increase the binding of DENV-2 to Vero cells (Figure 3(F)). We conclude that low pH enhances ZIKV infection, at least in part, by increasing the binding of viral particles to the cell surface. Because low pH has shown to induce irreversible changes in viral-surface proteins of a number of viruses in order to facilitate virus fusion with endosomal membranes [37–39], we analysed whether a transient exposure of ZIKV to low pH was able to promote viral infectivity. As shown in Figure 3(G), no changes in the infection pattern were observed when ZIKV was pre-incubated at low pH before Vero cell infection. Similar results were observed in assays performed in VK2/E6E7 cells (Figure 3(H)).

To further support the idea that low extracellular pH affects mainly ZIKV binding to cells, we performed a set of experiments exploiting the fact that NH_4_Cl inhibits the endosomal acidification necessary for viral fusion. We infected Vero cells in the presence of increasing concentrations of NH_4_Cl (2, 20, and 100 mM) at pH values of 7.3 or 6.2 and we found that even when viral fusion is being inhibited, low extracellular pH still induces an enhancement of viral infection, suggesting that acidosis exerts it’s effect upstream of viral fusion (Figure 3(I)).

In addition, as extracellular pH modification affects intracellular pH, we performed assays with NaHCO_3_-free media, taking advantage of the fact that NaHCO_3_ is necessary to equilibrate intracellular and extracellular pH. Interestingly, ZIKV infection was enhanced at pH 6.2 in NaHCO_3_-free media as it occurs in NaHCO_3_ rich media (Figure 3(J)). In parallel, we measured intracellular pH to show that in the absence of NaHCO_3_, extracellular pH variations do not modify intracellular pH. These data further point that acidosis in the extracellular compartment is responsible for enhancing ZIKV infection.

### Extracellular acidosis enhances ZIKV binding to cell surface heparan sulphate.

**Figure 4.**
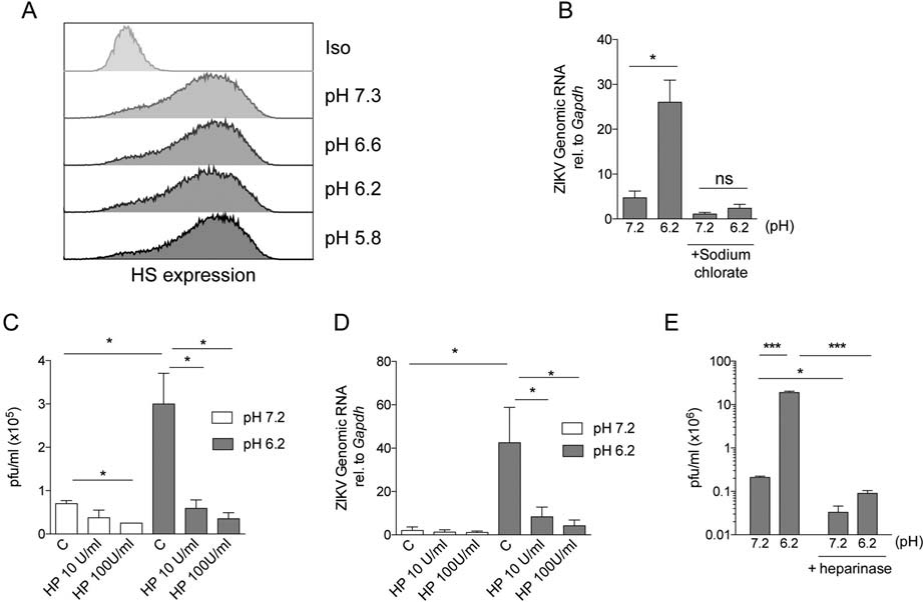
Enhancement of ZIKV infection by low pH depends on the expression of heparan sulfate by target cells. Vero cells were incubated for 1 h in pH adjusted media and 24 h later heparan sulfate expression was determined by flow cytometry (A). Vero cells were cultured for 3 days in sulfate-free media supplemented with sodium chlorate. Then, cells were challenged with ZIKV (A1C1-V2) for 1 h at 37°C, either at pH 7.3 or 6.2, and infection was analysed by qPCR 48 h later (B). Vero cells were challenged with ZIKV at pH 7.3 or 6.2 for 1 h at 37°C, in the absence (C: control) or presence of heparin (HP). Then, cells were washed and cultured for 48 h at pH 7.3 and infection was analysed (C). Vero cells were challenged with ZIKV at pH 7.3 or 6.2 for 1 h at 4°C, in the absence (C: control) or presence of heparin (HP). Thr binding of ZIKV to Vero cells was then analysed by qPCR (D). Vero cells were treated with heparinases for 2 h at 37°C and then infected either at pH 7.3 or 6.2. Forty-eight hours later infection was analysed by pfu assay (E). Results are expressed as the mean ± SEM of three independent experiments. ns, not significant.

Previous studies have shown that ZIKV interacts with cell surface heparan sulphate (HS) [29–31], enabling virus attachment to a variety of target cells including Vero [30], placental and brain cells [29]. To analyse whether the enhanced binding of ZIKV to target cells is dependent on HS expression, we performed a new set of experiments in conditions in which the expression of HS was inhibited [35]. First, we observed that HS surface expression in Vero cells was not modified by incubation for 1 h at low pH values compared to neutral pH (7.3) (Figure 4(A)). Then, Vero cells were cultured for three days in a sulphate-free medium supplemented with sodium chlorate, an inhibitor of sulphation. As we previously shown [40], this treatment resulted in a marked reduction (> 90%) in the expression cell surface HS (data not shown). These cells were incubated with ZIKV at pH 7.3 or 6.2 for 1 h, washed, and incubated for 48 h at 37°C. Then, infection was revealed by qPCR. A remarkable inhibition of infection was observed in cells challenged at low pH. In fact, under these experimental conditions, no differences were observed in the infection levels for cells challenged at either, pH 7.3 or pH 6.2 (Figure 4(B)).

Heparin has a similar molecular structure to HS and it is frequently used to assess the role of cell surface HS in viral infections. Heparin has been previously shown to inhibit the infection of different target cells by flaviviruses, including yellow fever virus, dengue, and Japanese encephalitis virus in Vero cells [41–43]. However, Young Kim et al. [30] demonstrated that heparin slightly promoted ZIKV replication, in a concentration-dependent manner in assays performed at neutral pH. We found that heparin slightly inhibited the infection of ZIKV particles to Vero cells at neutral pH while it exerted a dramatic inhibition of ZIKV viral production at low pH. Moreover, at pH 6, ZIKV binding to Vero cells was also inhibited by heparin. By contrast, heparin did not inhibit the binding of ZIKV to Vero cells at neutral pH (Figure 4(C,D).

To evaluate the specific role of HS and to distinguish it from other glycosaminoglycans, such as dermatan sulphate, Vero cells were first treated with a mixture of heparinases I and III [40,44], and then infected at pH 7.3 or 6.2. Enzymatic removal of HS with heparinases significantly prevented the infection when cells were infected at low pH (Figure 4(E)). Overall, our data show an important role for HS in ZIKV infection at low pH values.

## Discussion

We report for the first time that acidic values of pH markedly increase ZIKV infection in cells derived from the female genital tract, macrophages, and astrocytes. Consistently, low pH also increased ZIKV infection of human ectocervix and endocervix cultured ex-vivo. The effect imprinted by extracellular acidosis on ZIKV infection was shown to be strain independent as it was reproduced using three geographically different ZIKV isolates. Moreover, it was also observed using viral stocks obtained from either *A. albopictus* derived cell line C6/36 or Vero cells that differ in their glycosylation pattern [45]. Because the vaginal mucosa features low pH values, even after semen deposition [13,16,17], our observations suggest it represents a permissive environment for ZIKV infection. Of note, enhancement of infection by low extracellular pH was not observed for Dengue virus infection, another virus from the Flaviviridae family.

A previous study showed that ZIKV has the highest infectivity when viral particles were treated at pH 9 for 10 min before infection at neutral pH [46], however, under our experimental conditions, ZIKV infection was similar at pH values of 8, 9, and 7.3 (neutral pH) (data not shown). Moreover, this report shows that 10 min at pH 6 partially inactivates ZIKV viral stocks. Different times of exposition to pH and/or interaction with target cells at different pH could be explaining these contrasting results. It is also possible that this is a particularity of the strain (MR 766) the authors used.

The infectious cycle of ZIKV starts with its binding to cellular receptors and attachment factors. Several studies have revealed that ZIKV and DENV share various cell surface receptors, including Tyro3, DC-SIGN, and TIM-1 [47]. However, none of these proteins has been validated to be a unique and physiologically relevant receptor of ZIKV [48]. HS, a linear polysaccharide found in almost all tissues, has been identified as an important attachment factor for several flaviviruses, including DENV [41], JEV [27], and YFV [28]. The negative charges of the long carbohydrate chains of HS enable the interaction of HS with positive-charged groups expressed on the viral surface. In fact, HS is employed as a non-specific attachment factor by a number of viruses, including herpes simplex virus [26], Semliki forest virus [25], foot-and-mouth disease virus [24], Sindbis virus [23], Chikungunya virus [22], EV-A71 [21], human respiratory syncytial virus [20], Echoviruses [49], Rabies virus [19], Coxsackievirus [18], and Rift Valley fever virus [50]. Recently, it has been identified that HS also acts as an attachment factor in SARS-CoV-2 infection [51]. Particularly for ZIKV, using a surface plasmon resonance binding assay, Kim et al. [29] showed that the recombinant ZIKV envelope protein interacts with various glycosaminoglycans including HS. In contrast, Tan et al. [31] reported that ZIKV does not interact with heparin, a glycosaminoglycan that has similar protein binding properties to HS. Gao et al. also explored the role of HS in the interaction between ZIKV and host cells, using the CRISPR/Cas9 system to disrupt three key genes that encode proteins involved in the process of HS biosynthesis [32]. They showed that, unlike DENV-2, HS is not essential in the ZIKV entry but it appears to play a role in viral replication [32].

Our results suggest that low pH enhances ZIKV infection by favouring the interaction of viral particles with target cell HS. In fact, when target cells were cultured in conditions under which the synthesis of HS was inhibited or in the presence of heparin, the enhancing effect of low pH on viral infectivity was almost completely prevented. Moreover, the fact that a similar enhancing effect was observed when target cells and viral particles were cultured for 1 h at 4°C or 37°C suggests that low pH increases viral infectivity by promoting the binding of viral particles to the cell surface and not through energy-dependent processes such as viral fusion or endocytosis [52]. We hypothesized that low pH might increase the affinity of the ZIKV E protein to HS via protonation of histidine residues (pKa ∼ 6.5). In fact, low pH values (range 6.0–7.0) have shown to increase the binding of a variety of proteins, such as the prion protein, gp64 of baculovirus, GM-CSF, selenoprotein P, vascular endothelial growth factor, and the nonfibrillar form of a beta-amyloid peptide to HS via protonation of histidine residues [53–58]. The ability of low pH to increase the binding of viruses to target cells is not exclusive of ZIKV. We have previously reported that low pH enhances the binding of HIV-1 to spermatozoa and epithelial cells, but not to CD4+ T cells or dendritic cells [40,44].

The multistep process of membrane fusion that enables ZIKV to infect target cells is pH-induced [52]. Sun et al. [59] have demonstrated that conserved residues expressed in E protein were protonated under low pH conditions and played key roles in driving the fusion process. While low pH in endosomal compartments enables ZIKV fusion by inducing conformational changes on ZIKV E protein our results suggest that extracellular acidosis enhances the binding of the viral particles to the cell surface thus increasing viral infectivity. Together, our observations suggest that low values of pH similar to those found in the female lower genital tract after vaginal intercourse favours the sexual transmission of ZIKV infection.

## Supplementary Material

Figure_for_review_2.tif

Figure_for_review_1.tif
