# Supplementary figures and images for: Extracellular acidosis enhances Zika virus infection both in human cells and ex-vivo tissue cultures from female reproductive tract
Source: Emerg Microbes Infect. 2021 Jun 13;10(1):1169–79. doi: 10.1080/22221751.2021.1932606 (PMC8205022; doi:10.1080/22221751.2021.1932606)

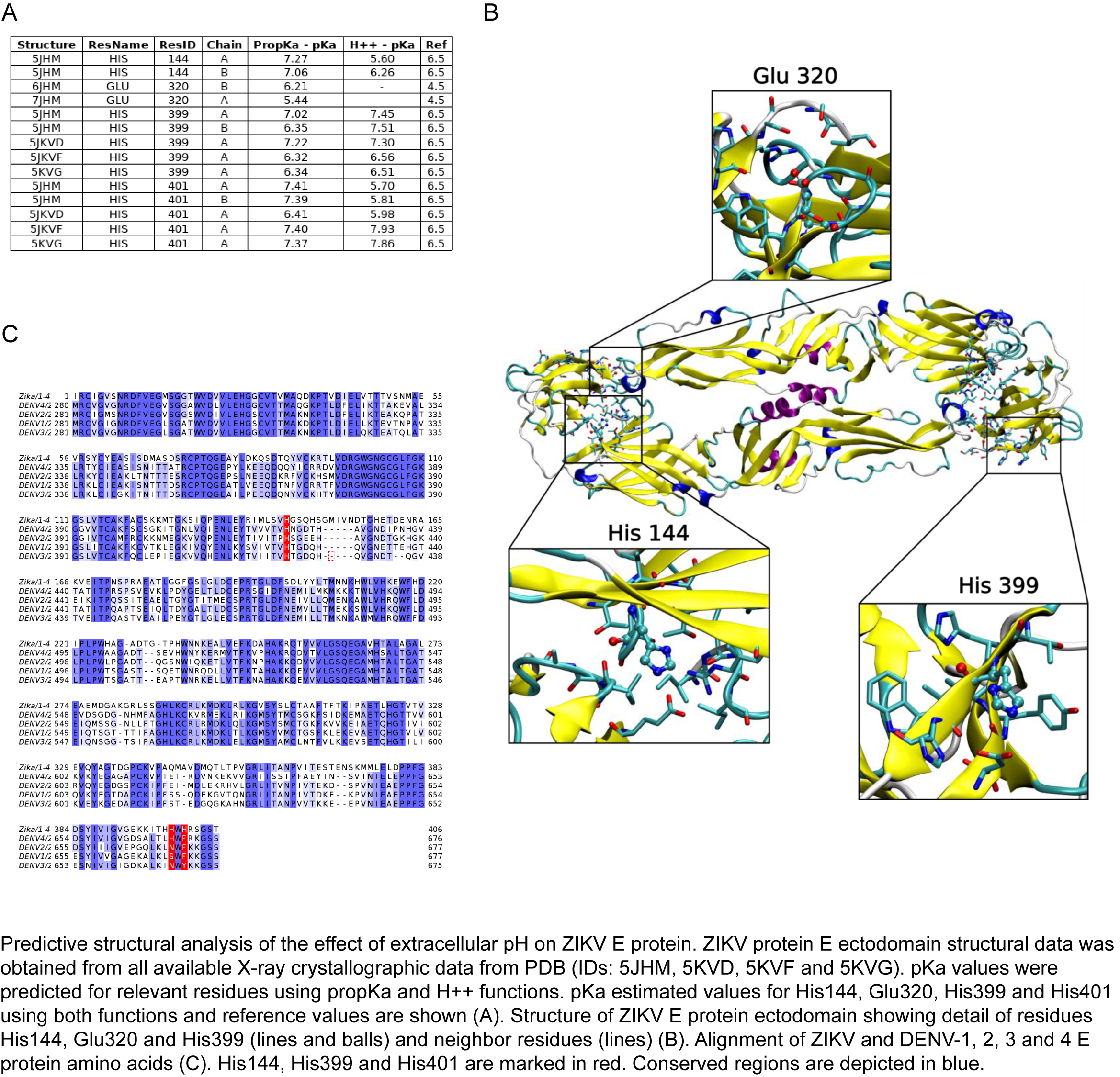

Supplement: Figure_for_review_2.tif [file TEMI_A_1932606_SM3977.tif]

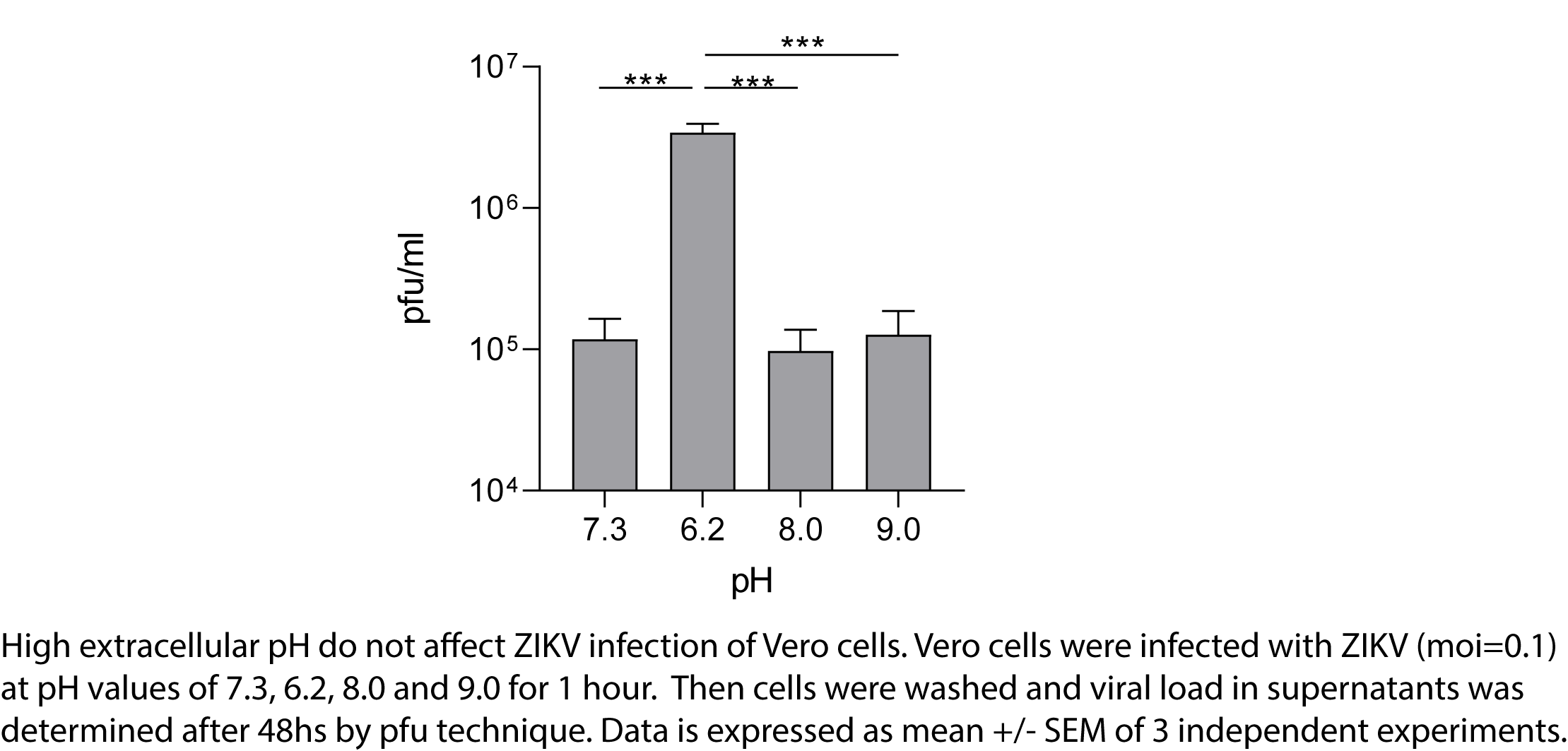

Supplement: Figure_for_review_1.tif [file TEMI_A_1932606_SM3976.tif]
